# 

**Published:** 1918-02-02

**Authors:** 


RATES OF SUBSCRIPTION: Twelve months, 6/6; Six months, 4/-; three months, 2/-, post free to any address in the United Kingdom. Abroad, Twelve months, 8/8; Six months, 5/-; Three months, 2/6, post free. **THE HOSPITAL "Index" to Volume LXII., April 1917 to September 1917, is now ready, price 2½d. per copy, post free.** Address The Manager, THE HOSPITAL, "The Hospital" Building, 28/29 Southampton Street, Strand, London, W.C. 2.

### THE WORK OF HOSPITAL ALMONERS

By **Miss A. E. CUMMINS** (Lady Almoner of St. Thomas' Hospital).

A useful and interesting guide to anyone
who desires to become a Hospital Almoner

Price 6d. net By Post, 7d

**The SCIENTIFIC PRESS, Ltd.**

**28/29 Southampton Street Strand, LONDON, W.C. 2**

### Recently Published Parliamentary Returns, etc.

National Health Insurance (Deposit Contributors' Benefits) Order, 1917. Post free 1½d.
Ditto, applied to Wales. Post free 1½d.
Physical Training in Secondary Schools in Wales. Post free 3d.
Midwives Act, 1902. Report on the Work of the Central Midwives Board for the year ended March 31, 1917. Post free 1½d.
Report on the Administration of National Health Insurance during the years 1914-17. Post free 1s. 11d.
Charity Commission. War Charities Act, 1916. Charities entered in the combined Register of Charities Registered in England and Wales under the Act up to December 31, 1917. Post free 2s. 11d.
Annual Report of the Local Government Board for Ireland for the year ended March 31, 1917. Post free 5d.
National Health Insurance. Transfer Values (Amendment) Regulations, 1917. Draft Regulations, December 28, 1917. Post free 1½d. Provisional Regulations, December 28, 1917. Post free 1½d.
Public Health, England. Prevention of Epidemic, Endemic, or Infectious Diseases. Public Health (Tuberculosis) Regulations, December 28, 1917. Post free 1½d.
Venereal Diseases. Provision for Diagnosis, Treatment, and Prevention. Circulars, etc., issued by the Local Government Board for Ireland. Post free 4d.

The above may be obtained from H.M. Stationery Office, Imperial House, Kingsway, W.C.

